# Variants in estrogen-biosynthesis genes *CYP17 *and *CYP19 *and breast cancer risk: a family-based genetic association study

**DOI:** 10.1186/bcr951

**Published:** 2004-11-11

**Authors:** Habibul Ahsan, Alice S Whittemore, Yu Chen, Ruby T Senie, Steven P Hamilton, Qiao Wang, Irina Gurvich, Regina M Santella

**Affiliations:** 1 Department of Epidemiology, Mailman School of Public Health, Columbia University, New York, New York, USA; 2 Herbert Irving Comprehensive Cancer Center, Columbia University, New York, New York, USA; 3 Division of Epidemiology, Department of Health Research and Policy, Stanford University School of Medicine, Stanford, California, USA; 4 Department of Psychiatry, Columbia University College of Physicians and Surgeons and the New York State Psychiatric Institute, New York, New York USA; 5 Department of Environmental Health Sciences, Mailman School of Public Health, Columbia University, New York, New York, USA

**Keywords:** breast cancer, cyp17, cyp19, estrogen biosynthesis genes, family-based design

## Abstract

**Background:**

Case-control studies have reported inconsistent results concerning breast cancer risk and polymorphisms in genes that control endogenous estrogen biosynthesis. We report findings from the first family-based association study examining associations between female breast cancer risk and polymorphisms in two key estrogen-biosynthesis genes *CYP17 *(T→C promoter polymorphism) and *CYP19 *(TTTA repeat polymorphism).

**Methods:**

We conducted the study among 278 nuclear families containing one or more daughters with breast cancer, with a total of 1123 family members (702 with available constitutional DNA and questionnaire data and 421 without them). These nuclear families were selected from breast cancer families participating in the Metropolitan New York Registry, one of the six centers of the National Cancer Institute's Breast Cancer Family Registry. We used likelihood-based statistical methods to examine allelic associations.

**Results:**

We found the *CYP19 *allele with 11 TTTA repeats to be associated with breast cancer risk in these families. We also found that maternal (but not paternal) carrier status of *CYP19 *alleles with 11 repeats tended to be associated with breast cancer risk in daughters (independently of the daughters' own genotype), suggesting a possible *in utero *effect of *CYP19*. We found no association of a woman's breast cancer risk either with her own or with her mother's *CYP17 *genotype.

**Conclusion:**

This family-based study indicates that a woman's personal and maternal carrier status of *CYP19 *11 TTTA repeat allele might be related to increased breast cancer risk. However, because this is the first study to report an association between *CYP19 *11 TTTA repeat allele and breast cancer, and because multiple comparisons have been made, the associations should be interpreted with caution and need confirmation in future family-based studies.

## Introduction

Cumulative exposure to circulating estrogen is considered to be of primary importance in breast cancer etiology. Estrogen biosynthesis, cellular binding and metabolism involve many steps, and the genes controlling these steps may contribute to inherent variability in breast cancer susceptibility. Endogenous estrogen is produced predominantly in the ovarian theca cells in premenopausal women and in the breast stromal adipose cells in postmenopausal women. The present study focuses on *CYP17 *and *CYP19*, two key genes that control the biosynthesis of estradiol and estrones from their lipid precursors and are expressed in these cells. CYP17 controls two successive early steps of endogenous estrogen biosynthesis by converting pregnenolone and progesterone to precursors of androgen and estrogen. CYP19, also known as aromatase, controls the terminal step of estrogen biosynthesis by converting 19-carbon steroids (testosterone and androstenedione) to 18-carbon estrogens (estradiol and estrone).

A T→C single-nucleotide polymorphism in the 5' promoter region of the *CYP17 *gene and a TTTA repeat polymorphism in the exon 4–intron 5 boundary region of the *CYP19 *gene have been investigated in breast cancer by several studies, with inconsistent results [1,2]. For both polymorphisms the variant alleles are considered to be related to an increased biosynthesis of endogenous estrogen. The *CYP17 *T→C polymorphism is thought to create an Sp1-type (CCACC) promoter site (although one study did not confirm this [3]) and is associated with an increased serum estrogen level [4,5]. After Feigelson and colleagues first published their study [6] showing a higher risk of breast cancer in relation to the *CYP17 *C allele among non-Caucasian women, many other authors attempted to replicate this in other populations. Although some studies confirmed this initial finding, others did not. All studies reporting an increased risk, including the original study, found the increased risk in one or more certain subgroups of women studied, for example women with advanced disease [6], women aged less than 40 years [7], women aged less than 40 years with family history [8], women aged more than 55 years [9], and women also carrying other genetic polymorphisms [10]. Two studies found that women carrying *CYP17 *C allele are less likely to use hormone replacement therapy [5,11] and three studies found that the protective effect of later age at menarche is stronger among women who do not carry the C allele [5,6,12]. A recent meta-analysis concluded that the *CYP17 *T→C polymorphism is not a significant independent risk factor for breast cancer [2].

The *CYP19 *gene contains a variable number (range 7–13) of TTTA repeats in the exon 4–intron 5 boundary region, creating polymorphisms that have been examined in five studies [13-17]. Kristensen and colleagues [13] and subsequently others found a roughly twofold to fourfold elevated risk in relation to certain numbers of *CYP19 *TTTA repeat polymorphisms. Although one small study found a higher risk in relation to the TTTA seven repeats allele, (TTTA)_7 _[14], most studies reporting an association found elevated risks in relation to one of the higher number of TTTA repeat alleles: 10 repeats, (TTTA)_12 _[13]; 12 repeats, (TTTA)_10 _[15,16]; or 10 or more repeats, (TTTA)_≥10 _[17]. A meta-analysis published in 1999 based on some of the earlier studies found that women carrying the *CYP19 *(TTTA)_10 _allele were at higher risk of breast cancer [1].

All published studies of association between the *CYP17 *and *CYP19 *polymorphisms and breast cancer discussed above used a classical case-control design. A recent meta-analysis of *CYP17 *T→C polymorphism indicates substantial differences in genotype frequencies in case-control studies conducted in different populations [2], with proportions of carriers ranging from 0.46 in the UK [18] to 0.79 in Japan [19] and proportions of homozygotes ranging from 11% in Finland [12] to 36% in Taiwan [10]. Similarly, the allele frequency of the *CYP19 *(TTTA)_10 _allele ranges from 0.5% [15] to 1.8% [14]. Given that case-control studies can be susceptible to population stratification bias, it is important to examine these potentially important biologically plausible hypotheses in family-based studies that are free from such bias. In this study we examine the association between the *CYP17 *promoter T→C and *CYP19 *TTTA repeat polymorphisms and female breast cancer by using a family-based design among nuclear families participating in the Metropolitan New York Registry (MNYR), one of the six international centers of the National Cancer Institute's Breast Cancer Family Registry project. Although other polymorphisms in the *CYP17 *and *CYP19 *genes have been reported, we focused on these two polymorphisms because they have been studied most extensively both in relation to their potential associations with breast cancer and also in relation to their influence on circulating estrogens.

All published studies focused on the relationship between a woman's own constitutional genotype and her breast cancer risk. A body of recent literature has provided limited data suggesting that a woman's breast cancer risk might be related not only to her own endogenous estrogens during adolescence and adulthood, but also to her prenatal exposure; that is, her exposure *in utero *to her maternal circulating estrogens [20-25]. In addition to the main association between a woman's own genotype and her breast cancer status, the family-based design of the present study allows us to address this hypothesis indirectly, by examining the association between maternal carrier status of *CYP17 *or *CYP19 *gene variants (that is, exposure *in utero *to an altered level of maternal estrogens) and breast cancer status in daughters.

## Methods

### Selection of study participants

Since 1995 the MNYR has been recruiting families with breast and/or ovarian cancers in clinical and community settings within the metropolitan New York area. Families meeting one or more of the following criteria are invited to participate: a female less than 45 years of age at diagnosis of breast cancer; a female with both breast and ovarian cancer; three or more relatives with breast or ovarian cancer diagnosed at age 45 years or more, or any male with breast cancer. After identification of a proband he/she is invited to participate in the registry and his/her family's eligibility is assessed. If the family is eligible and the proband agrees to participate, after appropriate informed consent, he/she is interviewed either in person or by phone with an epidemiology questionnaire and a family-history questionnaire. The proband is also asked to provide permission to contact family members. Blood or buccal samples are also collected and participants are provided with a self-administered dietary questionnaire to be returned by mail. Once family members consent to participate, data and blood or buccal samples from the family members are also collected in a similar manner. For members affected with cancer, tumor tissue samples are collected and reviewed pathologically. Genomic DNA from white blood cells or buccal samples has been collected for participants who donated biological samples. So far, the MNYR has enrolled 1158 families and more than 3900 total participants.

For this study we restricted attention to nuclear families with at least one affected daughter and at least one parent and/or sibling for whom DNA samples were available. Of the 1158 families enrolled in the MNYR so far, 278 families met these eligibility criteria. Subjects can participate in the MNYR with or without completion of the full epidemiology questionnaire and/or blood samples. There were 1123 family members in the 278 eligible nuclear families, of whom 702 completed the full epidemiology questionnaire and provided blood samples. However, accurate data on relevant variables for the statistical method used in this study (see below) for the remaining 421 members were available from the family-history questionnaire completed by the 702 members. There was 99% concordance in data on age and affected status between women who completed the full epidemiology questionnaire and women who did not.

### Laboratory analysis

We evaluated association between the T→C single-nucleotide polymorphism in the promoter region of the *CYP17 *gene and the tetranucleotide (TTTA) repeat polymorphism in the exon 4–intron 5 boundary of the *CYP19 *gene. A total of 23 subjects could not be genotyped for *CYP17*, and 26 subjects could not be genotyped for *CYP19*. Genotype data were available on a total of 679 members (from 277 nuclear families) for *CYP17 *and 676 members (from 278 nuclear families) for *CYP19*.

The *CYP17 *promoter polymorphism was determined with template-directed primer extension and detection by fluorescence polarization in a 96-microwell-based format [26,27]. In brief, DNA isolated from blood cells by salting out was used for genotyping subjects. First, the target DNA was amplified by polymerase chain reaction (PCR; using forward primer 5'-TTTAAAAGGCCTCCTTGTGC-3' and reverse primer 5'-TTGGGCCAAAACAAATAAGC-3') to generate products in the range 100–200 base pairs. After amplification by PCR, the primers were digested with shrimp alkaline phosphatase and *Escherichia coli *exonuclease I. Then single-nucleotide extension was performed in the presence of the appropriate allele-specific ddNTPs differentially fluorescence-labeled with either R110 or tetramethylrhodamine purchased from NEN Life Sciences (Boston, MA). For the single-nucleotide extension reaction both forward and reverse probes were tested to select the optimum (the forward probe 5'-GCCACAGCTCTTCTACTCCAC-3') on the basis of clear signal differences. The incorporation resulted in diminished rotation of the fluor compared with the ddNTP. Finally, the fluorescence polarization was read on a fluorescence polarization microplate reader (Tecan Polarion, Research Triangle Park, NC). The reader generates the genotype data on the basis of the distinct separations (with appropriate cut-offs) of the fluorescent intensity values for different alleles in comparison with internal controls.

The *CYP19 *TTTA repeats were determined by PCR amplification (using the forward primer 5'-GTCTATGAATGTGCCTTTTT-3' and the reverse primer 5'-GTTTGACTCCGTGTGTTTGA-3') followed by analysis on an ABI 377 system with GenScan software on the basis of the separations on gel according to the differences in the number of TTTA repeats.

All laboratory assays were performed with laboratory personnel blinded to the subject's disease status or family relationships. In addition to assay-specific quality-control samples, 10% of samples were reassayed after relabeling to keep laboratory staff blinded to its identity.

### Statistical analysis

We used the Family Genetic Analysis Program (FGAP [28], freely available at ) to test the null hypothesis of no association between genotype and breast cancer risk in nuclear families. The FGAP computes two test statistics: the nonfounder statistic (NFS), a generalization of the transmission disequilibrium test (TDT) [29,30], which evaluates transmission disequilibrium from parents to offspring, and the founder statistic (FS), which compares the distribution of parental genotypes with that expected under the null hypothesis of no association. The FGAP statistics fully exploit data from families with variable numbers of affected/unaffected members with variable (known/unknown) patterns of parental genotypes. They are similar to, but can be more powerful than, those available in the software FBAT [31]. (See [32] for a comparison of the methods.)

On the basis of the previous evidence [6,13,15,17], we hypothesized that breast cancer risk is elevated among carriers of the *CYP17 *C allele and the *CYP19 *variant alleles with 10 or more TTTA repeats, namely the (TTTA)_10_, (TTTA)_11_, (TTTA)_12_, and (TTTA)_13 _alleles. The data analysis was focused on two specific components of the study hypotheses: first, whether a woman's carrier status of the hypothesized alleles is associated with her breast cancer status, and second, whether a mother's carrier status of the hypothesized alleles is associated with her daughter's breast cancer risk. For testing the first component of a hypothesis, we applied the FS and NFS to assess whether specific genotypes of each of the studied genes are related to breast cancer. Because FS and NFS follow a normal Gaussian distribution under the null hypothesis, the assessment of statistical significance of the association can be done on the basis of the deviation of these statistics from the standard critical values under normal distribution.

For simplicity, we describe these analyses for the *CYP17 *gene as applied to nuclear families consisting of two parents and at least one daughter. Parents may be untyped and the mother's breast cancer status may be unknown. The test statistics, which are likelihood-based score statistics, are obtained by summing the score contributions from each family. These family-specific scores are obtained in three steps.

In the first step we imputed a probability distribution for the genotypes of each pair of parents, conditional on the observed genotypes of all family members. To do this, we obtained maximum-likelihood estimates of the genotypes TT, TC and CC for each of a pair of parents, given the observed genotypes in the family. These estimates do not require the assumption of Hardy–Weinberg frequencies for parental genotypes. If, for example, both parents' genotypes were known, then the probabilities are degenerate at the observed genotypes. Similarly, if both parents' genotypes were unknown but two offspring had observed *CYP17 *genotypes TT and CC, then the parental distributions are degenerate at TC because both parents must be heterozygotic.

In the second step we used the inferred parental genotype distribution and the offspring's observed genotypes to test whether heterozygous parents were equally likely to transmit T and C alleles to affected daughters. This evaluation is based on the NFS. Under the null hypothesis of equal transmission of T and C alleles from parents to affected daughters, the NFS has an asymptotic standard Gaussian distribution. The NFS generalizes the TDT to families with untyped parents and to families with both affected and unaffected daughters. It can be considerably more powerful than the sibling TDT test [33] when applied to families without unaffected daughters.

In the final step we used the inferred parental genotypes (and the mothers' breast cancer phenotypes) in the FS to compare the parental genotype distribution with the expected distribution under the null hypothesis of no association. This statistic treats the affected and unaffected mothers like cases and controls in a case-control study. However, each parent's contribution is weighted in proportion to his/her number of affected and unaffected daughters, so that parents of many affected daughters receive higher weights than do those of few affected daughters.

To test the second component of our hypothesis, namely the association between maternal carrier status and daughter's breast cancer status, we evaluated whether the genotypes of mothers with more affected daughters differ from those of mothers with less affected daughters. Such deviation might be expected if some aspect of a daughter's environment *in utero*, governed by the mother's genotype, influences the daughter's risk of subsequent breast cancer development. The FS was adapted to evaluate this question by comparing the observed or imputed genotypes of mothers of affected daughters with the genotypes expected in the parental population. It is a weighted sum of differences between each mother's observed (or inferred) C allele count and the average C count in the population. In symbols, each family's contribution to this sum is proportional to the quantity (*n*_A _- *n*_U_)(*C*_obs _- *C*_exp_), where *n*_A _and *n*_U _are,, respectively, the numbers of affected and unaffected daughters in the family, and *C*_obs _and *C*_exp _are the observed and expected C-allele counts for the mother. Under the null hypothesis of no association between maternal genotype and daughters' breast cancer risks, *C*_obs _has a mean value *C*_exp_, so *C*_obs _- *C*_exp _= 0 in expectation for all families. Thus the FS has expectation zero and the correct type I error rate regardless of the actual numbers of affected and unaffected daughters in each family. Under the alternative hypothesis that maternal C-allele count is associated with daughters' breast cancer risks, one expects that *C*_obs _- *C*_exp _> 0, and thus families with many affected daughters and few unaffected daughters (that is, *n*_A _- *n*_U _>> 0) contribute larger values to the FS than those with few affected daughters or those with many unaffected daughters. A statistically significant value of the FS when restricted to the mothers (with an insignificant value when restricted to the fathers) would provide evidence for this association.

When the null hypothesis is rejected, it is useful to estimate a measure of association between genotype and risk, such as the odds ratio, and to evaluate the effects of potential confounding by hormonal factors. To do so, we also performed conditional logistic regression analyses [34,35] on all the available sibships containing at least one affected sibling and at least one unaffected sibling who had provided blood samples and relevant epidemiology data for statistical adjustment (165 sibships for *CYP17 *and 169 sibships for *CYP19*).

## Results

Of the 277 nuclear families eligible for *CYP17 *analyses, 229 were Caucasian, 4 were African American, 41 were Hispanic, and 3 were Asian American. Of the 278 nuclear families eligible for CYP19 analyses, 229 were Caucasian, 4 were African American, 42 were Hispanic, and 3 were Asian American. Table 1 shows the distribution of the study subjects according to *CYP17 *and *CYP19 *genotypes, by family position and breast cancer status. The numbers in each cell represent the number of specific type of family members in our study population carrying a particular genotype. The number of TTTA repeats in intron 4 of the *CYP19 *gene ranged between 7 and 13 in our study population, with the (TTTA)_7 _and (TTTA)_11 _alleles being the most frequent (allele frequencies 53.9% and 28.8%, respectively). These frequencies are consistent with those found in Caucasian populations in other studies in the USA [15]. The frequency of the *CYP17 *variant C allele was 42.8% in this study population, which is similar to that found in other studies conducted in Caucasians [4].

The distribution of the nuclear families according to mother's and father's carrier status and mother's and daughter's affected status is presented in Table 2. A majority (about 55%) of the nuclear families contained one affected and one unaffected daughter. The majority of the nuclear families had one or more parents who did not have the genotyping information available.

Table 3 presents the FS and NFS for testing the associations between the *a priori *hypothesized *CYP17 *and *CYP19 *variant alleles and breast cancer. Each test statistic has an approximately standard Gaussian distribution under the null hypothesis of no association between genotype and breast cancer risk. A positive value of a NFS reflects excess transmission of the variant allele to affected daughters, and a negative value represents fewer such transmissions than expected under the null. Thus a test statistic that is negative but large in absolute value would suggest that the variant allele is associated with reduced risk. We computed the FS and NFS under recessive, dominant, and additive models. For the dominant models, the number of affected daughters carrying one or more copies of the variant alleles was compared with that expected from the parental genotypes in accordance with Mendelian expectation. Similarly, for the recessive models, the number of affected daughters homozygous for the variant allele was compared with that expected under Mendelian expectation. For the additive models, the total variant allele count in affected daughters was compared with that expected from the parental genotypes in accordance with Mendelian expectation. On the basis of the literature, we hypothesized *a priori *that *CYP19 *alleles with 10 or more TTTA repeats would be associated with breast cancer. In addition, we examined the association between the *CYP19 *genotype and breast cancer by defining the variant allele(s) by treating each of the 10 or more repeat alleles, (TTTA)_10_, (TTTA)_11_, (TTTA)_12 _and (TTTA)_13_, separately as the variant allele under each of the three models (realizing that this might have increased the chance of our finding of a statistically significant association; see the Discussion section).

As seen in Table 3, the NFS for association between the (TTTA)_11 _allele and breast cancer under the dominant model is 1.83, which is higher than the critical value (1.65) for a one-tailed test statistic, suggesting that affected daughters were more likely to receive the (TTTA)_11 _allele from their parents (irrespective of their ethnic distribution) than unaffected daughters. Like the NFS, the FS was also statistically significant under the dominant model, supporting an association between the *CYP19 *(TTTA)_11 _allele and breast cancer among the parents in these families. The results for *CYP19 *TTTA_≥10 _alleles did not show a consistent association, because only the FS was statistically significant under the dominant model. None of the other specific *CYP19 *alleles showed a consistent association with breast cancer on the basis of the NFS and FS (results not shown). Although the FS found an association between the *CYP19 *(TTTA)_13 _allele and breast cancer, this was not supported by the more robust NFS (results not shown).

Neither the FS nor the NFS suggested any significant association between the *CYP17 *variant C allele and breast cancer, under any of the models of FGAP analyses (see Table 3).

Table 4 presents the results of conditional logistic regression analysis comparing the *CYP17 *and *CYP19 *genotypes between affected and unaffected sisters. These results, adjusted for age (in years), hormone replacement use (ever/never), oral contraceptive use (ever/never), age at menarche (in years) and term pregnancies (yes/no), are similar to the FGAP results although because of the smaller number of available sibships the associations did not achieve statistical significance. As seen in Table 4, carriers of the *CYP19 *(TTTA)_11 _allele had an increased risk of breast cancer (odds ratio 1.8; 95% confidence interval 0.9–3.5).

Table 5 presents results relating maternal and paternal carrier statuses for the variants of estrogen-biosynthesis genes *CYP17 *and *CYP19 *to breast cancer risk in daughters. Mothers of affected daughters were more likely to carry the *CYP19 *(TTTA)_11 _allele than expected in the parental population. There were no such associations between the paternal carrier status of (TTTA)_11 _and any of the other *CYP19 *alleles and breast cancer in daughters. For this hypothesis, the findings for analysis involving *CYP19 *(TTTA)_≥10 _corroborated that for (TTTA)_11 _alleles. Although maternal carrier status of the *CYP17 *C allele tended to be positively associated with daughter's breast cancer, this association was not specific to the mothers but was also present among the fathers.

## Discussion

Despite a sound biological basis for the role of estrogen-biosynthesis genes in breast cancer, the findings of studies investigating the relationship between these genes and breast cancer have not been consistent. Employing a case-control design, many of these prior studies, especially those examining the *CYP17 *gene–breast cancer relationships, produced conflicting results. Although in comparison with *CYP17 *a smaller number of studies investigated the association of breast cancer with *CYP19*, findings for *CYP19 *have been more consistent, with most studies showing a positive association between *CYP19 *alleles with a higher number (10, 12, or 10 or more) of TTTA repeats and breast cancer [13,15-17].

Using a family-based design we investigated the relationships between the *CYP17 *and *CYP19 *gene variants and breast cancer in families participating in the MNYR. Like many of the previous case-control studies, the present study did not find any association between the *CYP17 *C (variant) allele and breast cancer. However, our findings support an association between certain alleles of the *CYP19 *intron 4 TTTA repeat polymorphism and breast cancer. On the basis of the previous studies we defined each of the *CYP19 *alleles with 10, 11, 12, or 13 TTTA repeats as the 'variant' allele and examined each association with breast cancer. Unlike some of the previous case-control studies we did not find the *CYP19 *(TTTA)_10 _or (TTTA)_12 _alleles to be associated with breast cancer. However, we found the *CYP19 *(TTTA)_11 _allele to be significantly associated with breast cancer in these nuclear families, under a dominant model. Although we also observed a significantly positive association between the *CYP19 *(TTTA)_13 _allele and breast cancer among the parents in these families, we did not observe excess transmission from parents to affected daughters, suggesting that the association might be due to chance or bias. The evidence of an increased risk in relation to the *CYP19 *(TTTA)_11 _allele was also observed in the conditional logistic regression analysis adjusting for potential confounding variables among the subset of families containing discordant sibships. However, because of the reduced power of these analyses among only a subset of families [36], results of these discordant sibship analyses did not achieve statistical significance.

In addition to evaluating associations between a woman's breast cancer risk and her own constitutional genotype, we also evaluated whether maternal genotypes are associated with the breast cancer risk in the daughters (independent of the daughter's own genotype). We found that the maternal (but not the paternal) genotypes of the *CYP19 *(TTTA)_11 _allele conferred a non-significantly elevated breast cancer risk to daughters. This effect was also observed when all (TTTA)_≥10 _alleles were treated as the variant allele. This association is consistent with evidence from the previous literature on the association between exposure to hormonal factors *in utero *and breast cancer risk in adulthood [20]. Although the association might be due to chance, if confirmed in subsequent studies it will have important implications in advancing our understanding of the breast cancer etiology.

Some limitations of the present study merit consideration. The major limitation concerns statistical power. The analysis, which is based on 287 nuclear families, might not have had enough power to detect small increases in risk associated with certain of the *CYP17 *genotypes. For example, we lacked power to evaluate interactions between genotypes for *CYP17 *and *CYP19 *and both endogenous and exogenous hormonal characteristics, such as age at menarche, timing and number of pregnancies and the use of exogenous hormones. In addition, although there is evidence for variations in the allele frequencies of the studied polymorphisms across ethnic groups, we lacked statistical power to conduct ethnicity-specific analyses. The evaluation of such analyses will be the subject of a separate future analysis, based on additional numbers of Breast Cancer Family Registry families.

Although the hypotheses examined in this study are not novel, the study design (which is free from population stratification bias) and the analytical approach have not been applied to these hypotheses in previous studies. Several limitations of this study require caution when interpreting the findings. First, the selection of nuclear families participating in this study from the MNYR was not population-based. Although this might limit the generalizability of the findings it should not affect the validity of the observed associations. Second, although it is possible for variations in the number of nucleotide repeats in hormone-related genes to be associated with cancer risk, such an association is less plausible biologically for the TTTA repeat numbers in the *CYP19 *gene. This is because the TTTA polymorphism is in the intronic region of the gene and so it is less likely that the variant alleles of the gene are directly associated with the functional status of endogenous estrogens in the body. Nevertheless, it is possible that one or more of the *CYP19 *TTTA alleles, including the (TTTA)_11 _allele, are in linkage disequilibrium with other functionally relevant alleles, as suggested by other studies [16]. Third, the present study compared multiple *CYP19 *TTTA alleles with breast cancer under different models. Although it is possible that multiple comparisons might have led to the observed associations, the consistency of the associations involving the *CYP19 *(TTTA)_11 _allele across both parents and transmission to offspring as well as the similarity between the associations with both constitutional and maternal genotypes suggest that these findings might have a biological basis. Further, the fact that the association was observed under specific susceptibility models and was consistent with conditional logistic regression analysis might be suggestive of the specificity of the finding.

## Conclusion

This family-based study found that the *CYP19 *(TTTA)_11 _allele is associated with breast cancer risk among families participating in a breast cancer family registry. The study also suggests that maternal carrier status of the *CYP19 *(TTTA)_11 _allele might be associated with breast cancer in daughters in these families. These associations might have important implications for understanding the etiology and risk prediction of breast cancer. However, because this is the first study to report an association with the *CYP19 *(TTTA)_11 _allele, and because multiple comparisons have been made, the associations reported in this study should be interpreted with caution and need to be confirmed in future family-based studies.

## Abbreviations

FGAP = Family Genetic Analysis Program; FS = founder statistic; MNYR = Metropolitan New York Registry; NFS = nonfounder statistic; PCR = polymerase chain reaction; TDT = transmission disequilibrium test.

## Competing interests

The author(s) declare that they have no competing interests.

## Figures and Tables

**Table 1:** Genotype distribution of the study population by gender, family relationship and affected status

	Mothers		Daughters				
				
Genotype	Affected	Unaffected	Affected	Unaffected	Fathers	Sons	Total
*CYP17*							
CC	2	10	47	41	6	11	117
CT	8	32	147	108	30	22	347
TT	2	19	107	61	8	18	215
Unknown	64	140	0	0	233	0	437
Total	76	201	301	210	277	51	1116
*CYP19 *(no. of TTTA repeats)							
7/7	1	15	88	55	7	21	187
7/8	0	10	34	29	4	5	82
7/9	0	0	1	0	1	0	2
7/10	0	1	5	6	2	0	14
7/11	9	19	106	71	14	19	238
7/12	0	2	9	8	0	0	19
7/13	0	0	0	0	1	0	1
8/8	0	0	4	1	0	0	5
8/10	0	1	3	2	0	0	6
8/11	1	2	24	16	1	3	47
8/12	0	0	1	2	0	0	3
8/13	0	0	0	1	0	0	1
9/11	0	0	0	1	0	0	1
10/11	0	0	2	3	0	2	7
11/11	0	5	19	16	1	1	42
11/12	0	1	4	2	0	0	7
11/13	1	0	2	1	1	0	5
11/not 11^a^	1	3	0	0	5	0	9
Unknown	63	143	0	0	241	0	447
Total	76	202	302	214	278	51	1123

^a^Indicates those whose genotype cannot be inferred for both alleles; the other allele could be 7, 8, or 12. Two of these nine observations, one an unaffected mother and the other the father in the same nuclear family, will be excluded when the allele with 10 or more repeats is selected as bad allele, because either them could be 11/12.

**Table 2:** Distribution of participating nuclear families according to mother's breast cancer status, mother's and father's carrier status of the *CYP17 *and *CYP19 *variant alleles, and number of affected and unaffected daughters

	Number of nuclear families according to mother's breast cancer status and genotype							Number of nuclear families according to father's genotype			
Number of daughters affected/unaffected	Affected			Unaffected			Total				Total
	Carrier	Non-carrier	Unknown	Carrier	Non-carrier	Unknown		Carrier	Non-carrier	Unknown	
*CYP17*											
1/0	3	0	19	17	12	29	80	12	2	66	80
1/1	5	2	38	16	6	85	152	19	4	129	152
1/2	0	0	2	4	0	14	20	4	2	14	20
1/3	0	0	1	2	0	0	3	0	0	3	3
2/0	2	0	3	1	1	9	16	0	0	16	16
2/1	0	0	0	0	0	2	2	0	0	2	2
2+/2+	0	0	1	2	0	1	4	1	0	3	4
Total	10	2	64	42	19	140	277	36	8	233	277

*CYP19 > = 10 repeats*											
1/0	4	0	17	14	13	29	77	7	4	66	77
1/1	5	1	40	12	9	88	155	10	6	139	155
1/2	1	0	1	5	1	14	22	5	2	15	22
1/3	1	0	0	1	0	0	2	1	0	1	2
2/0	1	0	4	1	1	9	16	1	0	15	16
2/1	0	0	0	0	0	2	2	0	0	2	2
2+/2+	0	0	1	0	1	2	4	0	0	4	4
Total	12	1	63	33	25	144	278	24	12	242	278

*CYP19 = 11 repeats*											
1/0	4	0	17	11	16	29	77	6	5	66	77
1/1	5	1	40	11	10	88	155	9	7	139	155
1/2	1	0	1	6	1	13	22	5	3	14	22
1/3	1	0	0	1	0	0	2	1	0	1	2
2/0	1	0	4	1	1	9	16	1	0	15	16
2/1	0	0	0	0	0	2	2	0	0	2	2
2+/2+	0	0	1	0	1	2	4	0	0	4	4
Total	12	1	63	30	29	143	278	22	15	241	278

**Table 3:** Association between the *CYP19 *and *CYP17 *variant alleles and breast cancer

		Nonfounder statistic			Founder statistic		
			
Variant allele(s)	Estimated allele frequency (%)	Recessive model	Dominant model	Additive model	Recessive model	Dominant model	Additive model
*CYP17*							
C	42.46	- 1.01	- 1.52	- 1.85	0.40	1.08	1.01
*P*		0.16	0.06	0.03	0.34	0.14	0.16
*CYP19*							
(TTTA)_≥10_	33.71	- 1.24	1.26	0.32	- 0.32	**1.66**	1.13
*P*		0.11	0.10	0.38	0.37	0.05	0.13
(TTTA)_11_	28.78	- 1.09	**1.83**	0.97	- 1.50	**1.96**	0.89
*P*		0.14	0.03	0.17	0.07	0.03	0.19

*P *values are based on one-tailed test statistics. Values that are statistically significant at one-tailed test are displayed in bold type.

**Table 4:** Conditional logistic regression analysis of discordant sibships for the association between *CYP17 *and *CYP19 *genotypes and breast cancer

Gene (sibling sets/cases/controls)		Affected (*n*)	Unaffected (*n*)	Adjusted odds ratios for breast cancer (95% CI)
*CYP17 *(165/171/188)				
Dominant model	TT	59	56	1.00
	TC/CC	112	132	0.86 (0.47–1.59)
Recessive model	TC/TT	146	154	1.00
	CC	25	34	0.61 (0.27–1.41)
General model	TT	59	56	1.00
	TC	87	98	0.86 (0.47–1.59)
	CC	25	34	0.55 (0.21–1.42)
Additive model (trend per allele)				0.77 (0.49–1.21)
*CYP19 *(no. of TTTA repeats) (169/175/193)				
Dominant model	(TTTA)_<10_(TTTA)_<10_	67	78	1.00
	(TTTA)_≥10_(TTTA)_<10_/(TTTA)_≥10_(TTTA)_≥10_	108	115	1.24 (0.63–2.46)
Recessive model	(TTTA)_<10_(TTTA)_<10_/(TTTA)_≥10_(TTTA)_<10_	159	173	1.00
	(TTTA)_≥10_(TTTA)_≥10_	16	20	0.82 (0.30–2.24)
General model	(TTTA)_<10_(TTTA)_<10_	67	78	1.00
	(TTTA)_≥10_(TTTA)_<10_	92	95	1.26 (0.64–2.51)
	(TTTA)_≥10_(TTTA)_≥10_	16	20	0.98 (0.30–3.18)
Additive model (trend per allele)				1.11 (0.65–1.89)
Dominant model	(TTTA)_other_(TTTA)_other_	77	95	1.00
	(TTTA)_11_(TTTA)_other_/(TTTA)_11_(TTTA)_11_	98	98	1.77 (0.90–3.47)
Recessive model	(TTTA)_other_(TTTA)_other_/(TTTA)_11_(TTTA)_other_	165	179	1.00
	(TTTA)_11_(TTTA)_11_	10	14	0.66 (0.19–2.33)
General model	(TTTA)_other_(TTTA)_other_	77	95	1.00
	(TTTA)_11_(TTTA)_other_	88	84	1.84 (0.93–3.63)
	(TTTA)_11_(TTTA)_11_	10	14	1.04 (0.27–4.08)
Additive model (trend per allele)				1.38 (0.79–2.40)

Odds ratios were adjusted for age (in years), hormone replacement use (ever/never), oral contraceptive use (ever/never), age at menarche (in years), full term pregnancies (yes/no). Each sibling set had at least one breast cancer case and one sister control. All the subjects included in the analysis had information for all the covariate variables. CI, confidence interval.

**Table 5:** Association between parental carrier status of the variant allele(s) and breast cancer risk in daughters

	Test statistic			
	
	Mothers' carrier status and disease risk in daughters		Fathers' carrier status and disease risk in daughters	
	
Variant allele(s)	Additive	Dominant	Additive	Dominant
*CYP17*				
C	1.47	1.09	1.40	1.07
*P*	0.07	0.14	0.08	0.14
*CYP19*				
(TTTA)_≥10_	1.73	1.65	0.95	0.45
*P*	0.04	0.05	0.17	0.33
(TTTA)_11_	1.52	1.96	- 0.11	0.18
*P*	0.06	0.03	0.46	0.43

The test statistic was calculated under the additive model. *P *values are based on one-tailed test statistics.
